# Silver Dimolybdate Nanorods: In Vitro Anticancer Activity Against Breast and Prostate Tumors and In Vivo Pharmacological Insights

**DOI:** 10.3390/pharmaceutics17030298

**Published:** 2025-02-24

**Authors:** João Victor Barbosa Moura, Natália Cristina Gomes-da-Silva, Luciana Magalhães Rebêlo Alencar, Wellington Castro Ferreira, Cleânio da Luz Lima, Ralph Santos-Oliveira

**Affiliations:** 1 Department of Physics, Federal University of Maranhão, São Luís 65080-805, MA, Brazil; luciana.alencar@ufma.br; 2 Laboratory of Nanoradiopharmacy and Synthesis of New Radiopharmaceuticals, Nuclear Engineering Institute, Brazilian Nuclear Energy Commission, Rio de Janeiro 21941-906, RJ, Brazil; nataliacristinasilva@id.uff.br; 3 Lyman Laboratory of Physics, Harvard University, Cambridge, MA 02138, USA; wcastroferreira@fas.harvard.edu; 4 Department of Physics, Federal University of Piauí, Teresina 64049-550, PI, Brazil; cleanio@ufpi.edu.br; 5 Laboratory of Radiopharmacy and Nanoradiopharmaceuticals, Rio de Janeiro State University, Rio de Janeiro 23070-200, RJ, Brazil

**Keywords:** cancer therapy, adjuvant therapy, nanoparticles, nanomaterials, oxidative stress, biodistribution

## Abstract

**Background**: The development of nanostructured materials for cancer therapy has garnered significant interest due to their unique physicochemical properties, including enhanced surface area and tunable electronic structures, which can facilitate targeted drug delivery and oxidative stress modulation. This study investigates the anticancer potential of monoclinic silver dimolybdate nanorods (m-Ag₂Mo₂O₇) against aggressive breast (MDA-MB-231) and prostate (PC-3) cancer cells and explores their in vivo pharmacokinetic behavior. **Methods**: m-Ag₂Mo₂O₇ nanorods were synthesized via a hydrothermal method and characterized using XRD, SEM, Raman, and FTIR spectroscopy. In vitro cytotoxicity was evaluated using MTT assays on MDA-MB-231 and PC-3 cell lines across concentrations ranging from 1.56 to 100 µg/mL. In vivo biodistribution and radiopharmacokinetics were assessed using technetium-99m-labeled nanorods in male Swiss rats, with gamma counting employed for tissue uptake analysis and pharmacokinetic parameter determination. **Results**: m-Ag₂Mo₂O₇ nanorods exhibited a modest cytotoxic effect on MDA-MB-231 cells, with 50 µg/mL reducing cell viability by 23.5% (*p* < 0.05), while no significant cytotoxicity was observed in PC-3 cells. In vivo studies revealed predominant accumulation in the stomach, liver, spleen, and bladder, indicating reticuloendothelial system uptake and renal clearance. Pharmacokinetic analysis showed a rapid systemic clearance (half-life ~6.76 h) and a low volume of distribution (0.0786 L), suggesting primary retention in circulation with minimal off-target diffusion. **Conclusions**: While m-Ag₂Mo₂O₇ nanorods display limited standalone cytotoxicity, their ability to induce oxidative stress and favorable pharmacokinetic profile support their potential as adjuvant agents in cancer therapy, particularly for chemoresistant breast cancers. Further studies are warranted to elucidate their molecular mechanisms, optimize combinatorial treatment strategies, and assess long-term safety in preclinical models.

## 1. Introduction

Nanostructured materials are emerging as pivotal tools in cancer treatment due to their unique physicochemical properties, such as their high surface area-to-volume ratio, tunable electronic structures, and enhanced interactions with biological systems at the molecular level [1,2]. These properties allow for improved drug loading, targeted delivery, prolonged retention, and enhanced therapeutic efficacy, reducing systemic toxicity and overcoming challenges like multidrug resistance and tumor heterogeneity [3,4,5]. Additionally, their ability to modulate reactive oxygen species (ROS) levels further enhances their anticancer potential, inducing oxidative stress selectively in cancer cells [4,5,6,7].

Molybdates, a class of transition metal oxides composed of MoO_x_ ions bonded to various cations, exhibit diverse morphologies, including nanorods, nanotubes, and nanoplates, which enhance their applicability in catalysis, drug delivery, and biological systems [8,9,10,11,12]. Molybdenum is an essential trace element involved in metabolic and detoxification pathways, and molybdate-based compounds have demonstrated promising anticancer effects by disrupting cancer cell metabolism, inducing apoptosis, and promoting oxidative stress responses [13,14]. Among these, silver molybdates (Ag_x_MoᵧO_x_) have gained attention for their antimicrobial and anticancer activities. Studies show that Ag_2_Mo_3_O_10_·2H_2_O nanorods and t-Ag_2_Mo_2_O_7_ microcrystals effectively inhibit bacterial strains, while silver molybdate (Ag_2_MoO_4_) has shown cytotoxicity against colon and hepatic carcinoma cells [15,16,17,18,19,20].

Despite their promising properties, the anticancer potential of molybdates remains underexplored. Research has indicated that sodium molybdate (Na_2_MoO_4_) can inhibit ovarian cancer growth through ferroptosis and apoptosis induction [21]. Similarly, gadolinium molybdate (Gd_2_(MoO_4_)_3_) doped with ytterbium has reduced breast cancer cell viability, while samarium molybdate (Sm_2_(MoO_4_)_3_) has demonstrated decreased proliferation in breast cancer cells [18,19,20].

Breast and prostate cancer are among the most prevalent cancers, with approximately 2.3 million new breast cancer cases and 1.4 million prostate cancer cases reported globally in 2020 [21,22]. The increasing incidence and challenges related to chemoresistance highlight the need for innovative therapeutic strategies. Nanostructured materials offer a multifaceted approach, integrating targeted drug delivery, ROS-mediated cytotoxicity, and photothermal therapy to improve treatment outcomes while minimizing damage to healthy tissues [4,5].

This study investigates the synthesis, characterization, and in vitro evaluation of m-Ag_2_Mo_2_O_7_ nanorods in aggressive breast (MDA-MB-231) and prostate (PC-3) cancer models. Given their high surface area, unique electronic properties, and known antimicrobial and anticancer potential, these nanorods hold significant promise as therapeutic agents. Furthermore, biodistribution and radiopharmacokinetic studies have been conducted to understand their biological behavior, clearance, and potential clinical applications. By combining innovative nanotechnology with oncology, this research aims to contribute to the development of next-generation nanomaterials for cancer therapy, addressing the urgent need for more effective and personalized treatments.

## 2. Materials and Methods

### 2.1. Reagents

The following culture reagents were purchased from Sigma-Aldrich (St. Louis, MO, USA): bovine serum albumin (BSA), penicillin, streptomycin, trypsin, Dulbecco’s Modified Eagle Medium (DMEM), trypan blue, The 3-(4,5-dimethylthiazol-2-yl)-2-5-diphenol tetrazolium bromide (MTT) assay, silver nitrite (AgNO_3_, 99.8%), sodium molybdate dihydrate (Na_2_MoO_4_·2H^2^O, 99.8%). The fetal bovine serum (FBS) was purchased from Cultilab (Campinas, SP, Brazil). 

### 2.2. Synthesis and Characterizations

The hydrothermal synthesis route obtained the m-Ag_2_Mo_2_O_7_ nanorods without the use of surfactants or organic additives. In a typical procedure [23], 1 mmol of silver nitrate [AgNO_3_] (99.8%, Sigma-Aldrich) and 0.5 mmol of sodium molybdate dihydrate [Na_2_MoO_4_·2H_2_O] (99.8%, Sigma-Aldrich) were diluted separately in 40 mL of deionized water. The AgNO3 solution was slowly added to the Na_2_MoO_4_·2H_2_O solution at room temperature and kept under intense stirring for 2 h. Then, still under stirring, nitric acid [HNO_3_] was added until it reached pH 2. The final solution was transferred to a Teflon-coated autoclave, which was closed and heated at 150 °C for 24 h. The formed precipitate was separated and washed by centrifugation using deionized water and ethanol. Then, the final material was dried in an oven at 60 °C for 12 h.

The structural characterization was conducted using X-ray diffraction (XRD) with a Bruker D8 Advanced diffractometer equipped with Cu Kα (λ = 1.5418 Å) radiation in the two-theta range of 10–90°. The data were collected with a step size of 0.02° and a count time of 1 s/step. Rietveld refinement [24] was performed using the FullProf software (version 3) [25] and crystal data were obtained from the Inorganic Crystal Structure Database (ICSD)—Card No. 154826. For the morphological analysis, samples were examined with a scanning electron microscope (SEM) model Vega3 Tescan. Raman measurements were conducted using a T64000 Horiba spectrometer equipped with a liquid N2-cooled CCD system. The 532 nm line of a solid-state laser was employed as the excitation source. An Olympus microscope lens with 100× magnification focused the laser beam onto the sample surface. The slit was set to a resolution of 2 cm^−1^. The spectrum was acquired through seven accumulations, with an acquisition time of 20 s. The Fourier-transform infrared (FT-IR) spectrum was measured in the spectral range of 4000–400 cm^−1^ on the spectrometer model Perkin Elmer Spectrum Two using a KBr pallet in the transmittance mode. The spectrum was recorded with 180 scans with a spectral resolution of 4 cm^−1^.

### 2.3. Cell Culture

Cell lines PC-3 (ATCC CRL-1435, a prostate tissue derived from a human) and MDA-MB-231 (ATCC HTB-132, a human breast cancer cell line) were obtained from the Cell Bank of Rio de Janeiro (Rio de Janeiro, Brazil). The cells were maintained in high glucose Dulbecco’s Modified Eagle Medium (DMEM- D5648, Sigma-Aldrich, USA) supplemented with 0.5 µg/mL penicillin and streptomycin (Gibico, São Paulo, Brazil), 5.2 g/L HEPES, sodium bicarbonate (3.7 g/L), 2 mM L-glutamine, 2.5 µg/mL fungizone, and 10% heat-inactivated FBS. The cells were incubated at 37 °C and 5% CO_2_ (CO_2_ incubator, series 3, Thermo Scientific, Waltham, MA, USA) until 70% confluence. The cells were subcultured in 25 or 75 cm^2^ culture flasks once a week and the culture medium was also changed once a week. The cells used in the experiments were from passages 04 and 29 for PC3 and MDA cells, respectively. The PC-3 and MDA-MB-231 cells were removed by trypsinization (0.1% trypsin/0.02% ethylenediamine tetraacetic acid, EDTA). The viable cells were counted in a Neubauer chamber under an optical microscope (100×).

### 2.4. Cytotoxicity Assessment and Inhibitory Concentration (IC_50_) Determination

The effect of m-Ag_2_Mo_2_O_7_ nanorods on cell viability was investigated using MTT reduction assays as described by Helal-Neto et al. [26]. The cell lines were incubated in 96-well plates (5 × 10^4^ cells/well) at 37 °C and 5% CO_2_ in the presence or absence of m-Ag_2_Mo_2_O_7_ nanorods (1.56–100 µg/mL). The toxicity of the m-Ag_2_Mo_2_O_7_ nanorods (1.56–100 µg/mL) was evaluated only by MTT. Two independent experiments were performed in quadruplicate. Blank (medium without cells), negative control (untreated cells), and positive control (DMSO) groups were included in each experiment to determine the No Observed Effect Concentrations (NOEC) and the IC_50_ (concentration that inhibits 50% of growth). After 24 h of incubation, MTT solution (final concentration of 1.0 mg/mL) was added to the wells. After 3 h at 37 °C and 5% CO_2_, the supernatants were discharged, and DMSO (Sigma-Aldrich, 200 µL/well) was added to solubilize formazan crystals. After 10 min, the absorbance was read in a microplate reader (Multiskan FC, Thermo Scientific) at 450 nm. Cell growth inhibition (%) was calculated according to the following equation, considering the absorbance or counts per minute values from the MTT reduction methods, respectively: % inhibition = 100 − (sample value × 100)/(untreated control). The viability (%) was calculated as the complement of the percentage of inhibition.

### 2.5. In Vivo Biodistribution: Tissue Deposition

#### 2.5.1. Labeling Process with 99mTc

The labeling process was performed using 150 µg of m-Ag_2_Mo_2_O_7_ incubated with stannous chloride (SnCl_2_) solution (80 µL/mL) (Sigma-Aldrich) for 20 min at room temperature. Then, this solution was incubated with 100 µCi (approximately 300 µL) of technetium-99m for another 10 min to label the structures.

#### 2.5.2. Quality Control of the Labeling Process with Tc-99m

To confirm the efficacy of the labeling process, radio thin-layer chromatography (RTLC) was performed using Whatman paper #1 and 2 μL of the ^99m^Tc-m-Ag_2_Mo_2_O_7_, along with acetone (Sigma-Aldrich) as the mobile phase, at times of 30 min, and 0, 0.5, 1, 3, 5, and 24 h. The radioactivity of the strips was verified in a γ-counter (Hidex, Turku, Finland). The RTLC was performed in triplicate for each time interval.

#### 2.5.3. Animals

Experiments were performed on male Swiss rats, *n* = 4, weighing between 20 and 30 g. Animals were housed one per cage under controlled conditions of luminosity (12:12 h light/dark cycle) and temperature (21.0 ± 1.0 °C), with free access to water and standard chow. All procedures were approved by the State University of Rio de Janeiro Animal Care and Use Committee (protocol number CEUA/8059100220/2021), which is consistent with the standards of the United States National Institute of Health Guide for Care and Use of Laboratory Animals (National Research Council, 1996).

#### 2.5.4. Animal Preparation

Animals were anesthetized by an intraperitoneal injection (ketamine 100 mg·kg^−1^ and xylazine 20 mg·kg^−1^).

#### 2.5.5. Design Protocol

For the biodistribution assay, 3.7 MBq/0.03 mL of ^99m^Tc-m-Ag_2_Mo_2_O_7_ was injected intraperitoneally (i.p.) to evaluate the systemic behavior in healthy animals. The mice were sacrificed 24 h post-injection with excess anesthesia (isoflurane chamber) and the organs of interest (heart, brain, stomach, intestine, bladder, kidneys (right and left), lungs (right and left), liver, spleen, and blood) were immediately separated and weighed for the quantitative estimation of gamma counts using a gamma counter (Hidex, Turku, Finland). The results were expressed as percentage of injected dose per organ (%ID/g).

### 2.6. Radiopharmacokinetics

For the radiopharmacokinetic studies, male Swiss mice (*n* = 4) received 4.1 mg per animal of ^99m^Tc-m-Ag_2_Mo_2_O_7_ (total activity of 90 µCi) administered by intraperitoneal injection. Subsequently, 2 µL of blood samples were collected from the tail vein at the following time points: 0, 2, 4, 6, 21, 23, and 24 h. The calculated pharmacokinetic parameters were as follows: (i) zero-time concentration (C_0_), (ii) elimination constant (K_e_), (iii) volume distribution (V_d_), (iv) clearance (CL), and (v) half-life elimination (t_1/2_). The radioactive count conversion to ^99m^Tc-m-Ag_2_Mo_2_O_7_ mass was calculated considering the initial mass of 4.1 mg using Equation (1).

(1) $$N=N_{o}e^{-ƛt}$$

### 2.7. Statistical Analyses

Viabilities are shown as the mean ± standard error of the mean (SEM). The half-maximal inhibitory concentration (IC_50_) and comparison of means (paired and multiple) were calculated using the GraphPad Prism 5 program (GraphPad Software version 8, La Jolla, CA, USA) considering significance to be at the level of *p* < 0.05. A paired *t* test was applied to investigate the similarity between the controls. Multiple comparisons of the data were performed using one-way analysis of variance (ANOVA) followed by Dunnett’s test (when the data were only compared to the negative control).

## 3. Results and Discussion

### 3.1. Characterization of Ag_2_Mo_2_O_7_ Nanorods

**Figure 1:**
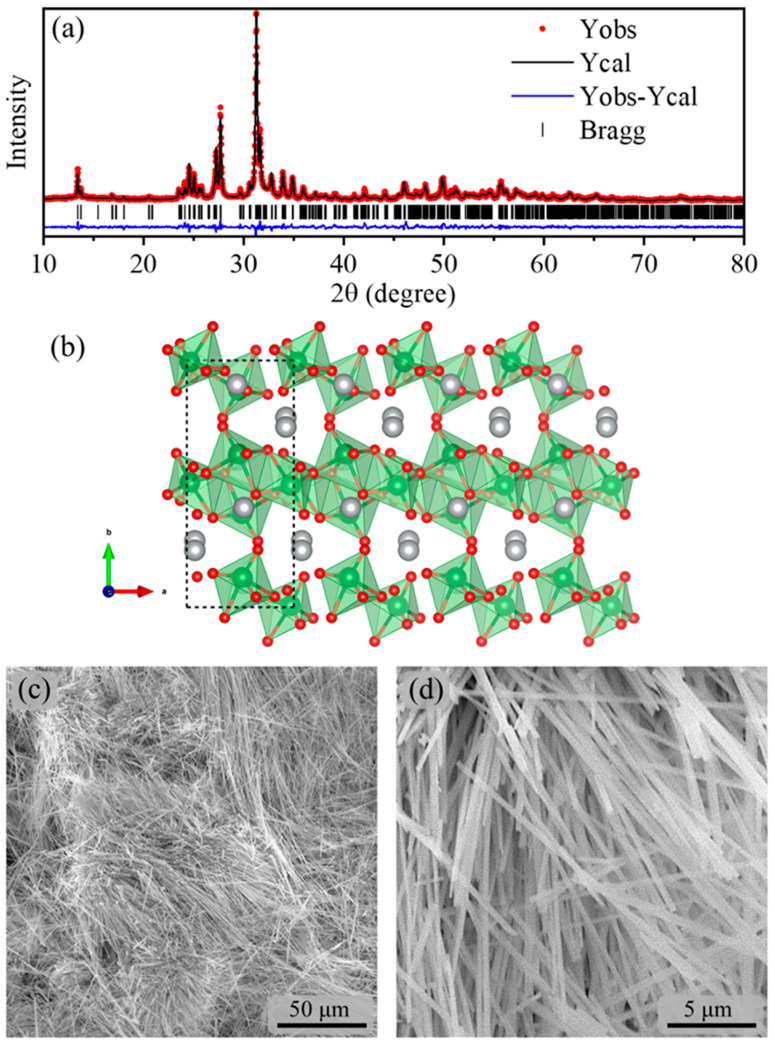
(**a**) X-ray diffraction pattern of m-Ag_2_Mo_2_O_7_ nanorods obtained by hydrothermal synthesis. (**b**) Representation of the crystal structure of the m-Ag_2_Mo_2_O_7_ crystal (Mo—Green, Ag—Silver, and O—Red atoms), showing the [MoO_6_] clusters connected by oxygen atoms forming distorted/deformed octahedrons. (**c**) Low- and (**d**) high-magnification SEM images of m-Ag_2_Mo_2_O_7_ nanorods.

The observed and calculated XRD patterns of the Ag_2_Mo_2_O_7_ nanorods are shown in Figure 1a. The intensity and sharpness of the diffraction peaks in the XRD pattern suggest that the as-synthesized Ag_2_Mo_2_O_7_ nanorods exhibited a well-crystallized structure. The Rietveld refinement yielded a good correlation between the observed and calculated XRD patterns, and no additional peaks were observed, thus confirming the phase purity of the Ag_2_Mo_2_O_7_ crystals. All the observed peaks could be accurately indexed to match the standard pure monoclinic phase of m-Ag_2_Mo_2_O_7_ (ICSD card No. 154826, space group P21/c). The quality indicators of the structural refinement, namely Rp = 4.57%, Rexp = 3.72%, Rwp = 5.96%, and χ^2^ = 2.57, further support the validity of the crystal phase. The calculated lattice parameters were determined to be a = 6.12416(26) Å, b = 13.18036(63) Å, c = 7.88691(35) Å, V = 595.463(46) Å^3^, and β = 110.71531(259)°. The obtained structural parameters are similar to the values reported in previous studies [23,27,28].

The lattice parameters and atomic positions, acquired through Rietveld refinement, were utilized to model the structure (see Figure 1b) using the Visualization for Electronic and Structural Analysis (VESTA) program [29]. The structure is formed by molybdenum (Mo) atoms coordinated by six oxygens forming distorted octahedral [MoO_6_] clusters connected by vertices and edges, giving rise to a polymeric 1D-[Mo_2_O_7_] ^2−^ block, a suitable topology to reach nanorod-like morphologies [28]. The morphologies of the as-synthesized monoclinic Ag_2_Mo_2_O_7_ samples were investigated using the recorded SEM images, as presented in Figure 1c,d. Figure 1 displays (c) low- and (d) high-magnification micrographs of Ag_2_Mo_2_O_7_, revealing a characteristic ultralong 1D rod with an average diameter of approximately 150 nm. The as-prepared material exhibited a high level of morphological homogeneity, free from any impurity particles, amorphous materials, or aggregates.

**Figure 2:**
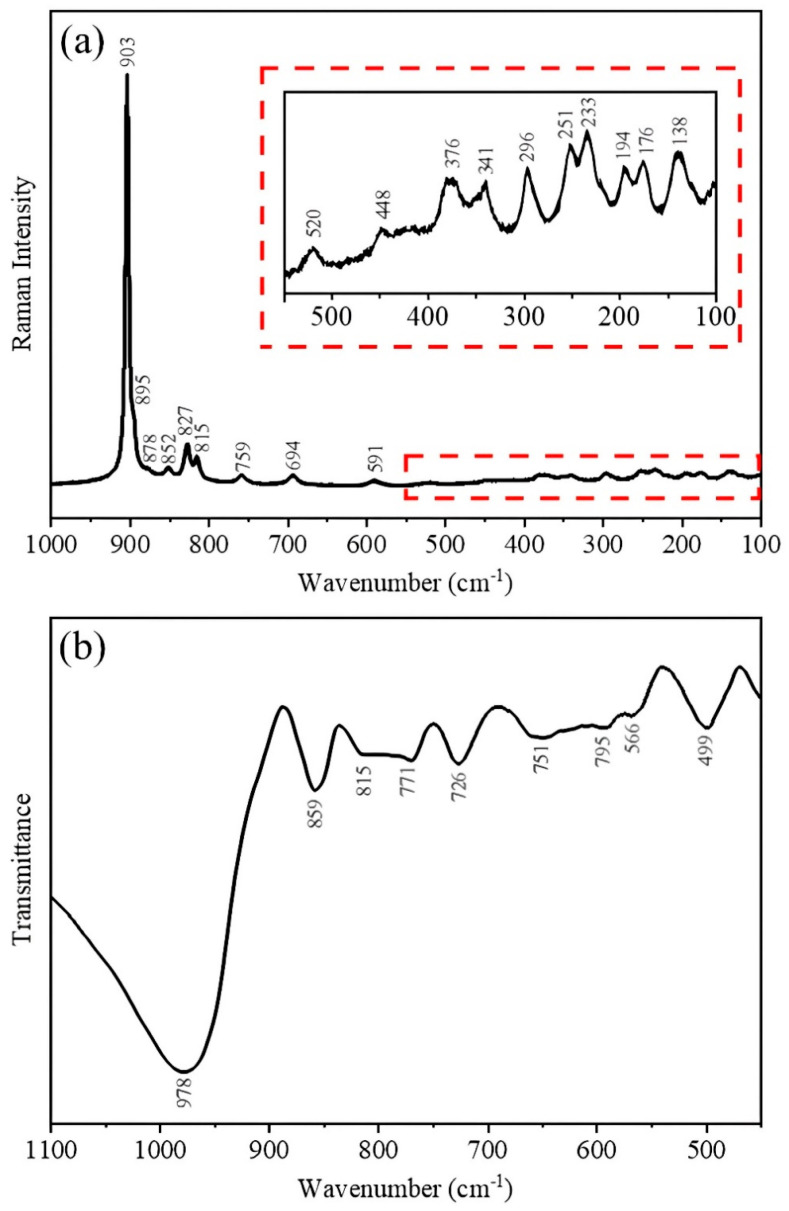
(**a**) Raman and (**b**) FTIR spectra of m-Ag_2_Mo_2_O_7_ nanorods. Inset: Magnification of the spectral region of 550–100 cm^−1^ where the Raman modes have low intensity.

The Raman spectrum of the m-Ag_2_Mo_2_O_7_ nanorods is presented in Figure 2a. The spectrum is divided into three sets of Raman modes: 1000–800 cm^−1^, 800–200 cm^−1^, and 200–80 cm^−1^. The Raman modes between 1000 and 800 cm^−1^ are associated with the symmetrical and asymmetrical stretching of Mo–O bonds in the MoO6 octahedra. In the region of 800–200 cm^−1^, the bending modes of O–Mo–O bonds are observed. Lastly, Raman modes below 200 cm^−1^ correspond to the translational/librational modes [23]. All of the Raman modes of m-Ag_2_Mo_2_O_7_ nanorods exhibit high intensity and well-defined peaks, suggesting that the samples are structurally ordered at a short range. The FTIR spectrum of m-Ag_2_Mo_2_O_7_ nanorods is shown in Figure 2b. The observed vibrational modes in the infrared spectrum correspond to the Mo=O and Mo–O–Mo bonds of the octahedral units, which are consistent with the findings reported by Hakouk et al. [28] and with the results obtained from the DFT calculations conducted by Yan et al. [30]. The vibrational spectroscopy results obtained by Raman and FTIR agree with the structural analysis performed by XRD and demonstrate success in synthesizing Ag_2_Mo_2_O_7_ nanostructures in the mono-clinic structure without secondary phases.

### 3.2. Cytotoxicity Effect on MDA-MB-231 and PC-3 Cells

**Figure 3:**
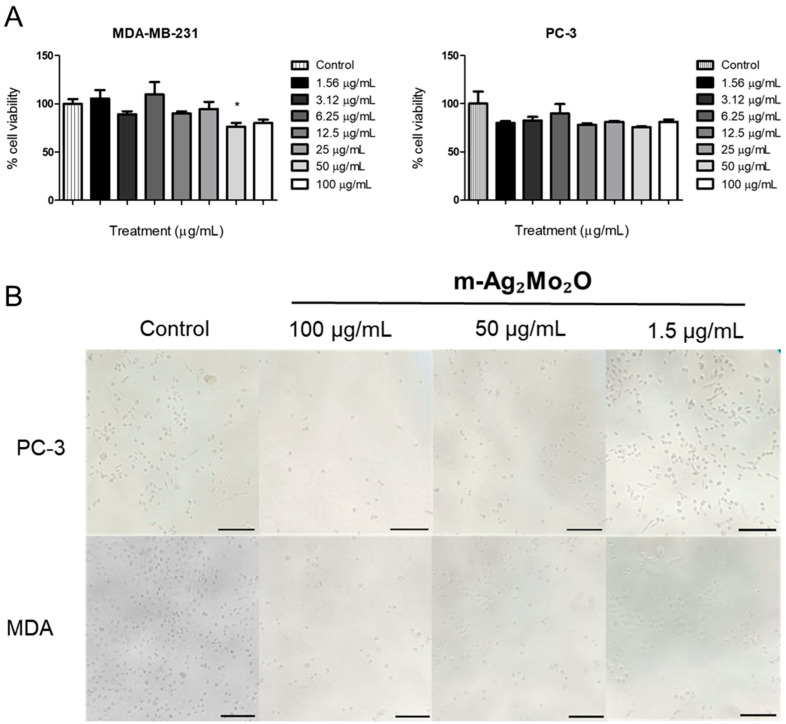
(**A**) MDA-MB-231 and PC-3: cell viability after treatment with different concentrations of m-Ag_2_Mo_2_O_7_ nanorods using a human breast cancer cell line and a prostate tissue derived from a human, respectively. The results are expressed as the percentage of total cell viability (spectrophotometric readings at 450 nm) in the culture medium after the addition of a 1 mg/mL MTT solution (positive response). The height of the histogram bar is the mean ± SEM of three independent experiments. MTT, 3-(4,5-dimethylthiazol-2-yl)-2,5-diphenyltetrazolium bromide. (**B**) The representative images of the key concentrations of m-Ag_2_Mo_2_O_7_ (control; 100 μg/mL; 50 μg/mL; and 1.5 μg/mL) obtained from an inverted light microscope with a 10× objective lens. Scale bar: 20 μm. * means statically different.

The cytotoxic effects of m-Ag_2_Mo_2_O_7_ nanorods on breast (MDA-MB-231) and prostate (PC-3) cancer cells were evaluated using seven concentrations (1.56–100 µg/mL) via the MTT assay (Table 1, Figure 3). After 24 h, 50 µg/mL exhibited a significant cytotoxic effect, reducing the MDA-MB-231 cell viability by 23.5% (*p* < 0.05). Although 100 µg/mL did not achieve statistical significance, a decreasing trend in cell viability (19.9%) was observed (Table 1). These results indicate that m-Ag_2_Mo_2_O_7_ nanorods have limited potency against these aggressive cancer cells at higher concentrations.

In contrast, no statistically significant cytotoxicity (*p* < 0.05) was observed for PC-3 and MDA-MB-231 cells across the tested concentrations. However, a slight reduction in viability was noted, particularly at 50 µg/mL, suggesting a modest cytotoxic effect. The reduction observed may stem from oxidative stress induction, metabolic disruption, or direct cellular interactions, warranting further mechanistic investigations.

It is important to notice however, that given the modest cytotoxicity observed for the m-Ag_2_Mo_2_O_7_ nanorods, their potential application as an adjuvant therapy in cancer treatment warrants consideration. Rather than serving as a standalone cytotoxic agent, these nanorods could be integrated into combinatorial therapeutic strategies to enhance the efficacy of existing chemotherapeutic or targeted treatments. Their ability to induce cell death suggests a potential role in sensitizing tumor cells to conventional drugs, particularly in cases of drug-resistant breast cancer, where oxidative stress modulation can enhance apoptosis and reduce tumor survival. Further studies should explore the synergy between the m-Ag_2_Mo_2_O_7_ nanorods and existing therapies, optimizing dosages and treatment regimens to maximize their therapeutic potential while minimizing toxicity.

Table 1 presents the viability of the adherent cell lines MDA-MB-231 and PC-3 following treatment with m-Ag_2_Mo_2_O_7_ nanorods at concentrations ranging from 1.56 to 100 µg/mL. In the MDA-MB-231 cell line, the m-Ag_2_Mo_2_O_7_ nanorods at 50 µg/mL exhibited cytotoxicity, reducing cell viability by more than 20% (23.5%, *p* < 0.05). Conversely, at 12.5 µg/mL, the nanorods were not cytotoxic to the PC-3 cell line (78.2–81.2% viability, *p* < 0.05), a trend that persisted across all tested concentrations.

The mechanism of the anticancer activity of m-Ag_2_Mo_2_O_7_ nanorods remains unexplored in the existing literature. However, previous studies on molybdenum-based nanoparticles (MoNPs) suggest that oxidative stress and reactive oxygen species (ROS) generation are likely key contributors to their cytotoxic effects [31,32,33]. For example, in a recent study, MoO_3_ nanoplates were evaluated in both human breast cancer cells (MCF-7) and non-cancerous keratinocyte cells (HaCaT) across a concentration range of 50–400 µg/mL [32]. Interestingly, only the cancerous MCF-7 cells exhibited a significant decrease in viability following MoNP treatment, while the non-cancerous HaCaT cells remained unaffected. Cytometric analysis suggested that this selective cytotoxicity involved apoptosis, likely triggered by the loss of mitochondrial membrane potential and ROS-induced oxidative stress.

Another study on L929 murine fibroblasts confirmed the dose- and time-dependent cytotoxic effects of MoNPs at concentrations ranging from 1 to 100 µg/mL using MTT and neutral red uptake (NRU) assays [34]. The oxidative stress induced by the nanoparticles was characterized by three key endpoints: lipid peroxidation, glutathione (GSH) depletion, and changes in catalase activity. These findings indicate that MoNP-induced oxidative stress compromises cellular antioxidant defenses, thereby leading to cell death.

Given these insights, it is plausible that the mild cytotoxicity of m-Ag_2_Mo_2_O_7_ nanorods in our study is similarly mediated through oxidative stress mechanisms, including ROS production, mitochondrial dysfunction, and GSH depletion. The selective cytotoxicity observed in cancer cells, such as MDA-MB-231 and PC-3, may arise from their heightened sensitivity to oxidative damage compared to normal cells. Future investigations should focus on exploring these potential mechanisms by evaluating ROS levels, the mitochondrial membrane potential, and antioxidant depletion following m-Ag_2_Mo_2_O_7_ nanorod treatment. Such studies would provide a more comprehensive understanding of the molecular pathways underlying the anticancer effects of these nanomaterials and confirm their potential for selective cancer targeting.

In this context, several interconnected aspects must be considered to clarify the factors that contribute to the low antitumor activity observed from m-Ag_2_Mo_2_O_7_ nanorods: (i) m-Ag_2_Mo_2_O_7_ has the ability to generate e^−^–h^+^ pairs since this material shows catalytic activity in the degradation of pollutants through this physical effect [27,35,36]. (ii) Analyzing the structural characterization of this material, it is observed that the Rietveld refinement results in the powder diffractogram (see Figure 1) indicate distortions in the octahedral sites formed by clusters of [MoO_6_], suggesting the potential for electron transfer and radiative transitions. (iii) Finally, another relevant piece of information is that the UV-Vis absorption spectra of m-Ag_2_Mo_2_O_7_ nanorods reported in the literature [35,36] reveal an optical bandgap value associated with intermediate energy levels between the valence and conduction bands.

Based on these findings, the mechanism underlying the anticancer effect can be attributed to the e^−^–h^+^ pairs formed by crystallographic defects in the material, which induce intermediate energy levels between the valence and conduction bands. Consequently, these species can react with H_2_O in an oxidation process, generating ^•^OH, H^+^, and O_2_^•−^ species. The generation of reactive oxygen species in a cellular system can induce damage by oxidative stress, the oxidation of biomolecules, and mitochondrial dysfunction, and can subsequently trigger necrotic and/or apoptotic cell death [37]. Indeed, tumor cells are more sensitive to external stimuli that further increase the production of ROS compared to normal cells, due to the already elevated ROS levels in tumors or their saturated antioxidant defenses [38,39,40].

This suggests that the unique electronic properties and structural features of m-Ag_2_Mo_2_O_7_ nanorods contribute to their potential as an effective strategy in fighting tumor cells. More investigations are needed to understand the action mechanisms and confirm the therapeutic potential of these nanorods in vivo. Furthermore, it is important to emphasize that more studies are needed to fully understand the efficacy, safety, and possible side effects of using m-Ag_2_Mo_2_O_7_ nanorods in cancer treatment.

### 3.3. In Vivo Biodistribution: Tissue Deposition

**Figure 4:**
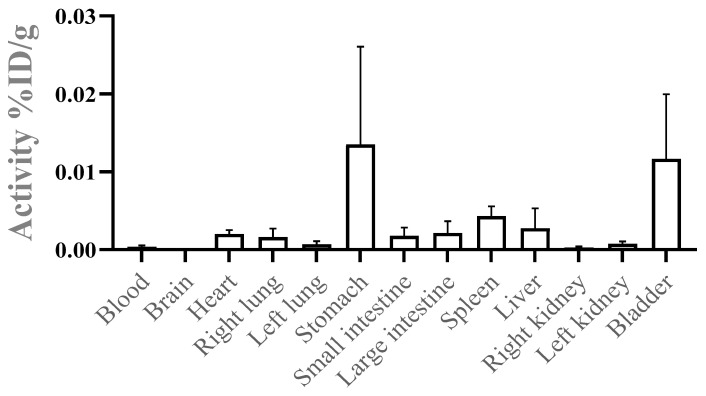
Biodistribution assay showing the tissue accumulation of m-Ag_2_Mo_2_O_7_.

The in vivo assay results, illustrated in Figure 4, reveal a significant uptake of m-Ag_2_Mo_2_O_7_ nanorods in the stomach, spleen, liver, and bladder. The elevated uptake in the stomach is likely a consequence of the intraperitoneal (i.p.) administration, which can cause the nanorods to adhere to the stomach lining, a phenomenon observed with other nanomaterials administered through this route.

As expected, the uptake in the liver and spleen can be attributed to the nanoscale dimensions of the crystals, which favor accumulation in the organs involved in mononuclear phagocyte system (MPS) activity. The liver and spleen play central roles in filtering foreign particles from circulation, including nanoparticles, through phagocytosis and endocytosis mechanisms.

The presence of nanorods in the bladder indicates renal clearance, suggesting that a portion of the material is effectively excreted through the urinary system. This observation is encouraging, as it demonstrates that the nanorods follow expected biological elimination pathways, minimizing the risk of prolonged retention and associated toxicity in non-target tissues.

These findings provide valuable insights into the biodistribution and clearance profile of m-Ag_2_Mo_2_O_7_ nanorods, reinforcing their potential suitability for therapeutic applications.

The significant uptake of m-Ag_2_Mo_2_O_7_ nanorods in the liver and spleen is consistent with the well-documented behavior of nanoparticles. These organs are central components of the reticuloendothelial system (RES), which efficiently captures nanosized particles from circulation. The particle size, surface modifications, and opsonization processes—where proteins in the bloodstream bind to nanoparticle surfaces—are key factors that influence this selective accumulation [41,42]. Smaller nanoparticles or those with hydrophilic surfaces are less likely to be retained by the RES, whereas larger or unmodified particles are preferentially sequestered by phagocytes within the liver and spleen.

The presence of m-Ag_2_Mo_2_O_7_ nanorods in the bladder suggests that renal clearance is a major elimination route, which is particularly relevant for the clinical application of nanoparticles. The size, shape, and surface chemistry of nanoparticles are critical factors in determining whether they can pass through the glomerular filtration barrier for urinary excretion [43,44]. Nanoparticles smaller than 6–8 nm typically undergo glomerular filtration directly, whereas larger nanoparticles may require degradation or surface modification to facilitate clearance through the kidneys.

Understanding the renal clearance mechanism is crucial not only for predicting the systemic circulation time of nanoparticles but also for assessing and mitigating potential nephrotoxicity. Ensuring efficient renal clearance minimizes the risk of prolonged nanoparticle retention in non-target tissues, which could otherwise lead to toxicity. Additionally, these insights open possibilities for renal-targeted drug delivery using m-Ag_2_Mo_2_O_7_ nanorods, making them a versatile platform for developing therapies targeting kidney-related diseases. Future investigations into pharmacokinetics and the potential bioaccumulation of these nanorods will provide further clarity on their safety and therapeutic utility.

### 3.4. Radiopharmacokinetics

**Figure 5:**
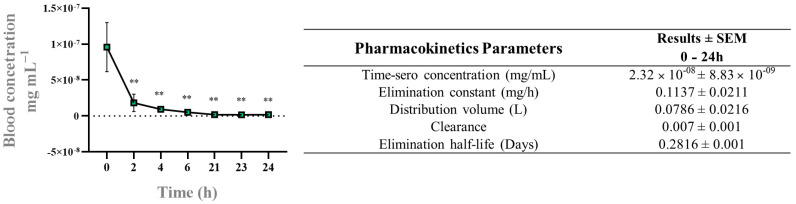
The main pharmacokinetic data derived from temporal reconstruction based on serial blood sampling. ** mean statically different.

The radiopharmacokinetics results (Figure 5) reveal key insights into the clearance, elimination, and distribution behavior of the m-Ag_2_Mo_2_O_7_ nanorods in vivo.

The renal clearance rate of 0.07 L/h indicates that 0.07 L of blood are cleared of the nanorods every hour, reflecting the combined efficiency of the excretion and metabolism processes responsible for removing the substance from the body. While this clearance rate is relatively low, it suggests that the nanorods are not eliminated rapidly, which could be advantageous for therapeutic applications requiring prolonged action, such as drug delivery systems. A slower clearance rate could allow the nanorods to remain in circulation long enough to exert sustained therapeutic effects before being excreted.

The elimination half-life of 0.2816 days (~6.76 h) suggests that m-Ag_2_Mo_2_O_7_ nanorods are cleared from the bloodstream relatively quickly. This half-life means that within approximately 6.76 h, the plasma concentration of the nanorods will decrease to half its initial value after administration. Although the clearance rate is not very high, the half-life indicates that elimination occurs within a manageable timeframe, likely due to a combination of renal clearance and metabolic breakdown [45,46]. For therapeutic applications, the dosing regimen would need to account for this rapid elimination to maintain effective plasma concentrations, either through frequent dosing or controlled-release formulations.

The volume of distribution (V_d_) of 0.0786 L provides insight into how the nanorods are distributed within the body. This relatively small V_d_ suggests that the nanorods are either confined largely to the blood plasma or have a high affinity for specific tissues or plasma proteins, limiting their movement into other compartments such as interstitial or intracellular fluids [47]. Such a limited distribution may be advantageous for therapies that target the vascular system or for the controlled release into circulation. Alternatively, the small Vd may also imply binding to plasma proteins, which keeps the nanorods localized and prevents extensive diffusion into peripheral tissues [48,49,50].

Together, these pharmacokinetic parameters highlight the need to optimize dosing strategies for effective therapeutic application. While the rapid elimination might require frequent dosing, the slow clearance offers the potential for prolonged therapeutic action. Furthermore, the small Vd suggests that the nanorods might be suited for systemic applications where the goal is to maintain drug concentrations in plasma rather than widespread tissue distribution. Understanding these properties will aid in developing precise dosing schedules and tailoring formulations to achieve optimal therapeutic outcomes.

## 4. Conclusions

This study demonstrates that monoclinic silver dimolybdate nanorods (m-Ag_2_Mo_2_O_7_) exhibit modest but selective cytotoxic effects against MDA-MB-231 breast cancer cells, with 50 µg/mL reducing the cell viability by 23.5% (*p* < 0.05) after 24 h. However, higher concentrations (100 µg/mL) did not show a statistically significant cytotoxicity, and the nanorods had limited efficacy against PC-3 prostate cancer cells, indicating that m-Ag_2_Mo_2_O_7_ is not a potent standalone cytotoxic agent.

Despite this, the ability of m-Ag_2_Mo_2_O_7_ nanorods to induce oxidative stress and potential mitochondrial dysfunction suggests a role as an adjuvant therapy. Given the challenges of chemoresistance in aggressive cancers, particularly triple-negative breast cancer, these nanorods may be used in combination with conventional chemotherapy to enhance the treatment efficacy through oxidative stress modulation and apoptosis induction. Future studies should focus on elucidating their precise mechanisms of action, potential drug synergy, and long-term safety profiles.

The in vivo biodistribution and radiopharmacokinetic data further support the feasibility of m-Ag_2_Mo_2_O_7_ nanorods for therapeutic applications. Their accumulation in the stomach, liver, spleen, and bladder, along with a clearance half-life of ~6.76 h, suggests rapid systemic elimination while maintaining a sufficient circulation time for therapeutic interaction. The small volume of distribution (0.0786 L) indicates limited diffusion into the peripheral tissues, making these nanorods a candidate for plasma-targeted drug delivery or controlled-release systems.

In conclusion, while m-Ag_2_Mo_2_O_7_ nanorods exhibit limited direct cytotoxicity, their unique physicochemical properties and biodistribution profile position them as a promising nanomaterial for adjuvant cancer therapy. Further investigations are necessary to optimize formulation strategies, explore combination therapies, and assess clinical feasibility in preclinical cancer models.

## Figures and Tables

**Table 1:** Cell viability (mean of IC_50_ ± SEM) at 1.56–100 µg/mL of m-Ag_2_Mo_2_O_7_ nanorods on cell lines assessed by MTT assays.

Method	Concentration (µg/mL)	Cell Viability (%)		Cell Death (%)	
		PC-3	MDA-MB-231	PC-3	MDA-MB-231
**MTT**	**100**	81.2 ± 2.4	80.1 ± 3.5	18.8 ± 2.4	19.9 ± 3.5
	**50**	75.5 ± 1.1	76.5 ± 3.8 *	24.5 ± 1.1	23.5 ± 3.8 *
	**25**	81.1 ± 1.2	94.6 ± 7.4	18.9 ± 1.2	5.4 ± 7.4
	**12.5**	78.2 ± 1.5	89.9 ± 2.2	21.8 ± 1.5	10.1 ± 2.2
	**6.25**	89.8 ± 9.8	109.8 ± 12.8	10.2 ± 9.8	ND
	**3.125**	82.6 ± 3.7	89.1 ± 3.2	17.4 ± 3.7	10.9 ± 3.2
	**1.56**	80.2 ± 1.6	105.6 ± 8.7	19.8 ± 1.6	ND
**Controls**	**SC**	100 ± 12.5	100 ± 5.0	---	---
	**DMSO**	ND	ND	---	---
**IC_50_**		>100	>100	---	---
**NOAEC**		>100	≥25	---	---

DMSO: positive control; SC: cell suspension, negative control; IC_50_: inhibitory concentration; NOAEC: No Observed Adverse Effect Concentration; ND: not determined. * Statistically significant decrease to control at *p* < 0.05 (ANOVA and Dunnett’s test).

## Data Availability

All data will be available under request.
